# Proteomic Differences in Feline Fibrosarcomas Grown Using Doxorubicin-Sensitive and -Resistant Cell Lines in the Chick Embryo Model

**DOI:** 10.3390/ijms19020576

**Published:** 2018-02-14

**Authors:** Katarzyna Zabielska-Koczywąs, Katarzyna Michalak, Anna Wojtalewicz, Mateusz Winiarczyk, Łukasz Adaszek, Stanisław Winiarczyk, Roman Lechowski

**Affiliations:** 1 Department of Small Animal Diseases with Clinic, Faculty of Veterinary Medicine, Warsaw University of Life Sciences, Nowoursynowska 159c, 02-787 Warsaw, Poland; aniawojt@hotmail.com (A.W.); roman_lechowski@sggw.pl (R.L.); 2 Department of Epizootiology and Clinic of Infectious Diseases, University of Life Sciences, Głęboka 30, 20-612 Lublin, Poland; epizootiologia@gmail.com (K.M.); lukasz.adaszek@up.lublin.pl (Ł.A.); stanislaw.winiarczyk@up.lublin.pl (S.W.); 3 Department of Vitreoretinal Surgery, Medical University of Lublin, Chmielna 1, 20-079 Lublin, Poland; winiarm86@gmail.com

**Keywords:** chemotherapy resistance, doxorubicin, feline injection-site sarcoma, in ovo assay, MALDI-TOF MS, proteomic analysis, two-dimensional electrophoresis

## Abstract

Proteomic analyses are rapid and powerful tools that are used to increase the understanding of cancer pathogenesis, discover cancer biomarkers and predictive markers, and select and monitor novel targets for cancer therapy. Feline injection-site sarcomas (FISS) are aggressive skin tumours with high recurrence rates, despite treatment with surgery, radiotherapy, and chemotherapy. Doxorubicin is a drug of choice for soft tissue sarcomas, including FISS. However, multidrug resistance is one of the major causes of chemotherapy failure. The main aim of the present study was to identify proteins that differentiate doxorubicin-resistant from doxorubicin-sensitive FISS using two-dimensional gel electrophoresis (2DE), followed by matrix-assisted laser desorption ionisation time-of-flight mass spectrometry (MALDI-TOF MS) analysis. Using the three-dimensional (3D) preclinical in ovo model, which resembles features of spontaneous fibrosarcomas, three significantly (*p* ≤ 0.05) differentially expressed proteins were identified in tumours grown from doxorubicin-resistant fibrosarcoma cell lines (FFS1 and FFS3) in comparison to the doxorubicin-sensitive one (FFS5): Annexin A5 (ANXA5), Annexin A3 (ANXA3), and meiosis-specific nuclear structural protein 1 (MNS1). Moreover, nine other proteins were significantly differentially expressed in tumours grown from the high doxorubicin-resistant cell line (FFS1) in comparison to sensitive one (FFS5). This study may be the first proteomic fingerprinting of FISS reported, identifying potential candidates for specific predictive biomarkers and research targets for doxorubicin-resistant FISS.

## 1. Introduction

Proteomic analyses are rapid and powerful tools that expand knowledge within cancer research. The proteomic profiling of biological material obtained from cancer patients (blood, urine, tumours) can be used to increase the understanding of cancer pathogenesis, discover proteomic cancer biomarkers, and select and monitor novel targets for cancer therapy [1,2,3]. The most well-known and widely used proteomic technology focuses on the large-scale, high-throughput separation and subsequent identification of proteins resolved by two-dimensional polyacrylamide gel electrophoresis. Mass spectrometry (MS) identification enables researchers to identify thousands of proteins that represent complex biosystems, as well as differences in the proteomic profiles between two different samples [4]. It is a modern method for a rapid and more sensitive high-throughput detection and identification of both known and unknown proteins [5]. In human medicine, proteomic analyses were successfully applied to identify proteins that are differentially expressed in malignant and benign human breast cancers [6,7], differentiate various breast cancer subtypes [8], and select biomarkers for tumour sensitivity for different chemotherapeutic agents [9,10]. In veterinary oncology, there are only a few studies on the application of matrix-assisted laser desorption ionisation time-of-flight mass spectrometry (MALDI-TOF MS) applied for canine B cell lymphomas, prostate and bladder carcinomas, mammary gland carcinomas, and cutaneous mast cell tumours [11,12,13,14,15,16]. According to the literature, in feline oncology MALDI-TOF MS analysis has only been described to differentiate the proteomic profile of lymph nodes from healthy cats and those with lymphoma [17]. The proteomic profiling of doxorubicin-sensitive and doxorubicin-resistant fibrosarcomas enlarge the molecular knowledge on feline injection-site sarcomas (FISS) chemoresistance, and may be a first step for the targeted therapy of FISS.

FISS are aggressive skin tumours with high recurrence rates ranging from 38–78%. Radical surgery, radiotherapy, and/or chemotherapy are used to prolong disease-free intervals or disease-free survival in cats. Nevertheless, the response rate varies around 50% [18]. Multidrug resistance (MDR) is one of the major causes of chemotherapy failure.

MDR is a multifactorial phenomenon, defined as the acquisition of the simultaneous insensitivity of tumour cells to several groups of different, unrelated therapeutic agents. It develops in response to the use of a single cytostatic drug [19]. Some neoplasms have primary resistance, which means that there is a minor response to cytotoxic drugs from the beginning of the treatment. Other tumours acquire it during chemotherapy treatment, which is called secondary resistance [20]. Both types of resistance mechanisms are connected to a change in the drug absorption intensity and the transport between the nucleus and cytoplasm, a change in the number and affinity between the enzymes and cytostatics, the activation or deactivation of pharmacological substances in the neoplastic cells, the ability to impede the regulation of apoptosis, changes in the reconstruction process of the DNA, as well as the active extraction of drugs from the neoplasm. The most commonly studied causes of resistance of the tumours to the cytostatics are the adenosine triphosphate-binding cassette (ABC) transporters [21]. Their task is to extract the cytostatics from the cytoplasm, thus protecting the neoplasm from their effects. Multifactorial resistance can also be shown through the gene expression analysis using the reverse transcription-polymerase chain reaction (RT-PCR) method or functional tests of the transmembrane efflux pumps, for example, the rhodamine 123 test and verapamil [22,23,24]. Recently, it has been indicated that proteome analyses more directly reflect cellular function than gene and transcript analyses [8]. In human medicine, MALDI-TOF MS has been utilised to identify proteins as potential biomarkers for chemotherapy responses in cervical cancer [25] and cutaneous melanoma [26].

The chick embryo chorioallantoic membrane (CAM) model is a preclinical in vivo model that follows the “3R” (reduction, replacement, refinement) guidelines, which need to be strictly followed after the implementation of directive 2010/63/EU on the protection of animals used for scientific purposes. The CAM model is widely used for angiogenesis assays, acute toxicological studies, and studies of neoplastic cell extravasation, bone regeneration, and molecular cancer biology [27,28,29,30,31,32,33,34]. In veterinary medicine, it has been applied in canine osteosarcoma, canine soft-tissue sarcoma, canine mammary gland tumors, feline mammary carcinoma, and feline fibrosarcoma research [35,36,37,38,39]. In the case of FISS, the CAM model has been shown to closely resembles spontaneous FISS, making it a good alternative for expensive, time-consuming rodent models, which as opposed to the CAM model, require Animal Ethics Commission approval [40].

The main aim of the present study was to identify proteins that differentiated doxorubicin-resistant from doxorubicin-sensitive FISS using the CAM model, which resembles features of spontaneous fibrosarcomas.

## 2. Results

### 2.1. In Ovo Assay

Twenty-two tumours were included in proteomic analyses (seven each from FFS1 and FFS3 cell lines, and eight from the FFS5 cell line). Two chicken embryos died on the seventh day of incubation due to manual manipulation. Fibrosarcoma tumourigenicity was 78.5% (22/28). Differences in protein expression levels between feline fibrosarcomas grown from doxorubicin-resistant (FFS1, FFS3) and doxorubicin-sensitive (FFS5) cell lines were identified using two-dimensional gel electrophoresis (2DE) and MALDI-TOF MS.

### 2.2. Protein Identification by 2DE and MALDI-TOF MS Analysis

**Figure 1:**
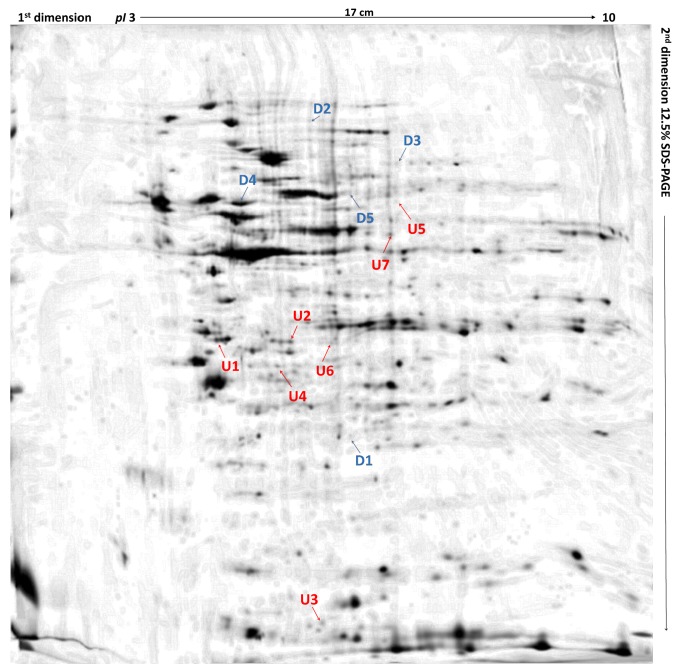
Differentially expressed proteins in feline fibrosarcomas from doxorubicin-resistant and doxorubicin-sensitive cell lines. U1 and U2, up-regulated proteins in both FFS1 versus FFS5 and FFS3 versus FFS5; D1, down-regulated protein in both FFS1 versus FFS5, and FFS3 versus FFS5; U3–U7, up-regulated proteins in FFS1 versus FFS5; D2–D5, down-regulated proteins in FFS1 versus FFS5. Up-regulated proteins are indicated in red, and down-regulated proteins are indicated in blue. Merged two-dimensional electrophoresis gels of representative tissue lysates of tumours from FFS1, FFS3, and FFS5 cell lines. Proteins were separated in the first dimension by isoelectric focusing over the isoelectric point (pI) range 3–10. The second dimension was performed using 12.5% sodium dodecyl sulfate polyacrylamide gel. Gels were silver stained, digitised, and processed in Delta2D software (version 4.7 DECODON Greifswald, Germany).

**Figure 2:**
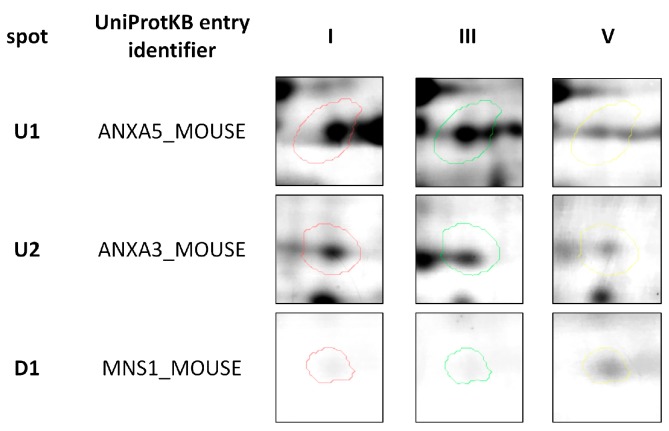
Representative two-dimensional electrophoresis gels of significantly (*p* ≤ 0.05) differentially expressed proteins in feline fibrosarcomas from both the FFS1 cell line versus the FFS5 cell line, and the FFS3 cell line versus the FFS5 cell line.

Three out of a total of 260 proteins detected on all of the gels were identified as significantly differentially expressed (*p* ≤ 0.05) in doxorubicin-resistant fibrosarcomas (from both FFS1 and FFS3 cell lines) when compared to doxorubicin-sensitive fibrosarcomas (from the FFS5 cell line) (FFS1/FFS5 and FFS3/FFS5 ratio >1.5 or <0.67, *p* ≤ 0.05) (Table 1; Figure 1). Two proteins: Annexin A5 (ANXA5) and Annexin A3 (ANXA3) were up-regulated, and the meiosis-specific nuclear structural protein 1 (MNS1) was down-regulated (Figure 2). Most of the cases with top-scoring results were obtained from mouse (*Mus musculus*), and a few from human (*Homo sapiens*), which can be easily explained by the limited cat sequences available in the database used for the search (SwissProt, www.uniprot.org). There were several proteins of *Gallus gallus* shown in the MS identification report, but they were not significantly differently expressed, with an MS score below 40, or much lower in comparison to *Mus musculus* or *Homo sapiens*.

**Figure 3:**
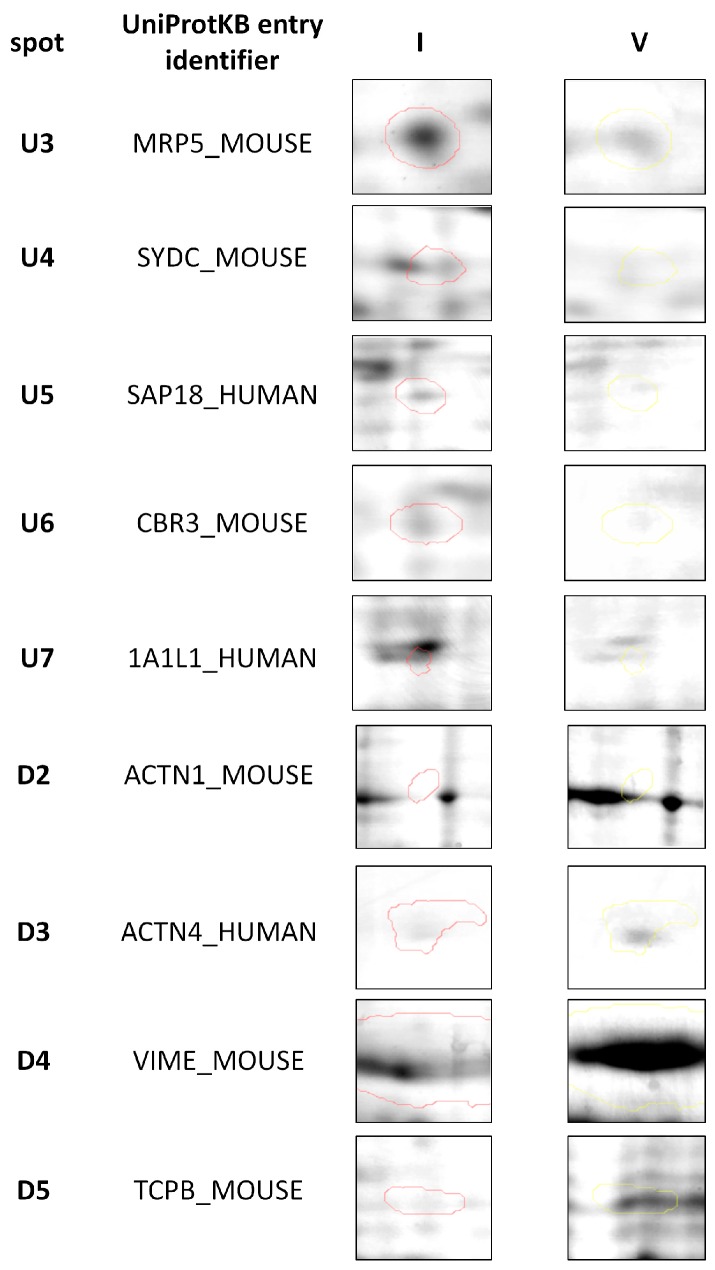
Representative two-dimensional electrophoresis gels of proteins that were significantly (*p* ≤ 0.05) differentially expressed in feline fibrosarcomas from the FFS1 cell line versus the FFS5 cell line.

Moreover, when comparing tumours from the only high doxorubicin-resistant cell line (FFS1) to those from the doxorubicin-sensitive cell line (FFS5), nine additional proteins were significantly differentially expressed (Table 2), indicating the proteins that may play role in tumours with high primary doxorubicin resistance. Five of them were up-regulated, and four were down-regulated (Figure 3).

## 3. Discussion

Most proteins identified by MALDI-TOF MS analyses have been described in the context of MDR, although only a few of them concern doxorubicin-resistance. Both ANXA5 and ANXA3 lead to the expression of the annexin family of calcium dependent phospholipid-binding and membrane-binding proteins. ANXA5 has a strong affinity for phosphatidylserine, which is present on the cell membrane, and is a hallmark of cell death that is understood not only by apoptosis and necrosis, but also by mitotic catastrophe, autophagy, and senescence [41]. In human medicine, ANXA5 overexpression has been associated with carcinogenesis (e.g., in cervical carcinoma, breast cancer, fibrosarcoma, osteosarcoma, and prostate cancer), cancer cell invasion, metastasis (e.g., in squamous cell carcinoma and colorectal cancer) and drug resistance (e.g., in gastric cancer, nasopharyngeal carcinoma, glioblastoma multiforme, and large B cell lymphoma) [42,43,44,45,46,47]. The overexpression of ANXA5 increased the half maximal inhibitory concentration (IC50) values of temozolomide in glioblastoma multiforme cell lines, indicating its role in acquired drug resistance. Also, in a nasopharyngeal carcinoma cell line, the up-regulation of ANXA5 was suggested as a possible cause of resistance for cis-diamminedichloroplatinum vincristine, carboplatin–taxotere, and 5-fluorouracil. Yoshida et al., pointed out ANXA5 as a possible candidate for the doxorubicin resistance of both primary and induced doxorubicin-resistant lung cancer cell lines [45]. This is in agreement with the findings presented in this study, which showed a significantly (*p* ≤ 0.05) higher expression of ANXA5 in the doxorubicin-resistant FISS model in comparison to the sensitive one. ANXA3 is another cytosolic protein that exhibits an important role in tumourigenesis and metastasis; in either, its expression depends on the tumour type [48]. The up-regulation of ANXA3 promoted the development of colorectal adenocarcinoma [49] and pancreatic carcinoma [50], and increases the metastatic risk of lung adenocarcinoma [51] and hepatic carcinoma [52]. While on the other hand, decreased ANXA3 expression was negatively correlated with the progression of prostate and renal carcinoma [53,54]. There is only one study on the role of ANXA3 in chemotherapy resistance, which indicates that the up-regulation of ANXA3 enhanced platinum resistance in ovarian carcinoma [55]. Our findings from the present study demonstrated the up-regulation of ANXA3 in tumours from both FFS1 and FFS3 cell lines, suggesting that it may be associated with drug resistance.

The significantly lower expression of MNS1 in all of the tumours that were grown from doxorubicin-resistant cell lines in comparison to the doxorubicin-sensitive cell line indicates its role in primary MDR. Little is known on the role of MNS1 in cancer research and chemoresistance, as it is a coiled-coil protein of unknown function expressed in the pachytene stage of the spermatogenesis, in the diplotene spermatocytes, and in the spermatids that are essential for spermatogenesis [56,57]. Nevertheless, a recent study on the proteome profiling of kidney fibroblasts showed MNS1 expression only under hypoxic conditions, and suggested that it is related to the early stages of meiosis and involves changes in its nuclear organisation [58]. The possible connection between gametogenesis and cancer development has been previously suggested; for example, the expression of the synaptonemal complex protein 1 (SCP1), a protein selectively expressed during the meiotic prophase of the spermatocyte, has been reported in several tumour types, including: glioma, breast cancer, and melanoma, suggesting that it may contribute to genomic instability [59]. The sperm protein 17 (Sp17), which was primarily thought to be restricted only to developing spermatozoa and mature spermatids, has been shown to be expressed in breast cancer, multiple myeloma, epithelial ovarian cancer, and esophageal cancer, making it a potential candidate target for immunotherapy [60,61]. The *MNS1* gene was shown to be down-regulated in an oxaliplatin-resistant human colon cancer cell line, and up-regulated in an oxaliplatin-sensitive human colon cancer cell line after continuous treatment with oxaliplatin for 24 h, suggesting its molecular role in chemoresistance [62]. MNS1 down-regulation in doxorubicin-resistant fibrosarcomas indicates that the protein plays a role in MDR for various chemotherapeutics with different mechanisms of action. The up-regulation of ANXA5 and ANXA3 may be correlated with drug resistance, while the down-regulation of MNS1 correlates with drug sensitivity in FISS. This study appears to be the first proteomic fingerprinting of FISS, and could serve as the first step in identifying possible candidates for specific predictive biomarkers and new research targets for doxorubicin-resistant FISS. To confirm this hypothesis, further studies on large samples taken from cats should be two-fold. (1) First, they should confirm the molecular predictive markers of FISS through performing proteome analyses on feline fibrosarcoma samples in vivo, and correlating them with clinical outcomes. (2) Second, they should reveal the mechanism of chemotherapy resistance in FISS in relation to identified proteins through in vitro studies on feline fibrosarcoma cell lines treated with various chemotherapeutic agents. Nevertheless, further studies, including semi-quantitative immunohistochemical analyses of tumors and intact tissue, and the assessment of the functional role of differently expressed proteins through knock-down in tumor cell isolates and their response to doxorubicin treatment, are needed to confirm this hypothesis.

From the nine identified proteins between high doxorubicin-resistant and doxorubicin-sensitive FISS, some of them (multidrug resistance protein 5 (MRP5), α actin 1 (ACTA1), α actinin 4 (ACTN4), vimentin (VIME), T-complex protein 1 subunit β (TCPB)) were previously reported as possible targets for MDR or indicators of anticancer drug chemosensitivity. MRP5, which is a homologue of MRP1, confers resistance to 5-fluorouracil. Yoshida et al., demonstrated the higher expression of MRP5 in doxorubicin-resistant human lung cancer cell lines, as well as its overexpression due to 48 h of exposure to doxorubicin, indicating its role in both intrinsic and acquired doxorubicin resistance [45]. Similarly, the results of the present study demonstrated the significantly higher expression of MRP5 in primary high doxorubicin-resistant FISS in comparison to doxorubicin-sensitive ones, which supports its role in intrinsic doxorubicin resistance. MRP5 may be a molecular predictive marker or treatment target for FISS. However, further studies are needed to confirm this hypothesis.

ACTN4 is a non-muscle isoform of α actinins that belong to the spectrin superfamily. It is expressed only in non-muscle cells, where they mediate actin filament bundling and interact with the cell membrane [63]. The correlation between ACTN4 expression and tumour grade, tumour progression, and metastasis were reported for various human cancers, including osteosarcoma, melanoma, leukemia, prostate cancer, bladder cancer, lymphoma, pancreatic cancer, breast cancer, and gastric cancer [63,64]. ACTN4 is not only responsible for cell motility, it also protects the cell from mechanical stress. In relation to its role in chemoresistance, both the high immunoexpression of the ACTN4 protein, and a high number of *ACTN4* gene copies, detected by fluorescence in situ hybridisation (FISH), positively correlate with chemoresistance (polychemotherapy with cyclophosphamide, doxorubicin, and cisplatin) of high grade ovarian cancers. When comparing immunohistochemistry with FISH, the FISH technique had better accuracy [65]; thus, due to its high specificity, MALDI-TOF MS may be used as a good alternative for ACTN4 detection. Also, significant differences in ACTN4 expression are suggested to play a role in chemoresistance in breast cancer; either its up-regulation or down-regulation varies depending on the chemotherapeutic. After treatment with tamoxifen and taxotere/carboplatin +/− Terceptim, ACTN4 was significantly up-regulated and down-regulated, respectively [66,67]. The results of this study indicate that primary high doxorubicin-resistance significantly down-regulates the expression of ACTN4.

VIME is a type-III intermediate filament protein that is expressed in mesodermal cells, epithelial cells, and leukocytes, as well as in various malignant tumours with epithelial origin. The proteomic data characterising its role in chemotherapy resistance varies between the studies, chemotherapeutic agents, and types of cancer. In human colon cancer, doxorubicin resistance is associated with the acquisition of an epithelial–mesenchymal transition, triggering the expression of VIME, *N*-cadherin, and Snail and Slug expression in resistant cells (HCT 168) [68], while in MDR human breast cancer (resistant to taxotere, epirubicin, and cyclophosphamide), VIME was significantly down-regulated [69]. He et al., also showed that the overexpression of VIME in human breast cancer is an indicator of a favourable response to chemotherapy [67]. These results are supported by the findings within this report, as tumours highly resistant to doxorubicin were characterised by a significantly lower expression of VIME.

TCPB, which serves as a molecular chaperon by assisting the folding of proteins upon the adenosine triphosphate (ATP) hydrolysis, is known to play a role in actin and tubulin formation in vitro [70]. Although this study presented TCPB down-regulation in high doxorubicin-resistant FISS, there is only one other study presenting its down-regulation in TS1-resistant pancreatic cancer cells, which indicates their role in chemosensitivity for TS1—a novel oral anticancer agent containing two biochemical modulators for 5-fluorouracil (5-FU) and tegafur, a metabolically activated prodrug of 5-FU [71].

The advantages of using the chicken embryo model of FISS for proteomic fingerprinting rather than FISS cell cultures include that it is the in vivo 3D model that, in some cases, represents tumour heterogeneity that cannot be observed in vitro. It is also an easy-to-implement model that follows the “3R” (reduction, replacement, refinement) guidelines and resembles spontaneous FISS [40].

## 4. Materials and Methods

### 4.1. Cell Lines

Three feline fibrosarcoma cell lines: doxorubicin-resistant (FFS1 and FFS3, high and intermediate resistance, respectively) and doxorubicin-sensitive (FFS5) with previously assessed pg-P activity [72] were routinely maintained in Dulbecco’s modified Eagle’s Medium (DMEM) with glucose enriched with 10% (*v*/*v*) heat-inactivated fetal bovine serum, penicillin-streptomycin (50 IU·mL^−1^), fungizone (2.5 mg·mL^−1^); they were then incubated in a humidified incubator at 37 °C with 5% CO_2_. All reagents were obtained from Gibco Brl, Life Technologies GmbH, Karlsrahe, Germany. All of the experiments were performed when cells reached 75–80% confluence. The viability and number of cells for in ovo assay were assessed with the Invitrogen Countless II Automatic Cell Counter (Thermo Fisher Scientific, Darmstadt, Germany).

### 4.2. In Ovo Assay

Fertilised eggs (*Gallus gallus*) (*n* = 30) were incubated at 37 °C and 70% humidity in an incubator according to the previously described protocol [40]. Briefly, on the sixth day of incubation, chick embryos were divided into three groups (*n* = 10 per group) and injected with 5 × 10^6^ fibrosarcoma cells from the FFS1, FFS3, or FFS5 cell line, and then placed on a CAM silicon ring. On the 16th day of incubation, the chicken embryos were euthanised by decapitation, and the tumours were collected and directly snap-frozen in liquid nitrogen. Frozen samples were rinsed twice with physiological saline (0.9% NaCl) and homogenised in tris(hydroxymethyl)aminomethane (TRIS) buffer (1.5 M, pH 8.8) with the addition of a protease inhibitor cocktail (Sigma Aldrich, Poznań, Poland). Obtained homogenates were centrifuged at 13,000 rpm for 20 min at 4 °C. Supernatants were collected and centrifuged once more under the same conditions. The homogenates obtained were transferred into 0.5 mL microcentrifuge tubes and stored at −80 °C.

### 4.3. Protein Cleaning and Precipitation

Protein concentration was measured by the spectrophotometric method (MaestroNano Micro-Volume Spectrophotometer, MAESTROGEN, Hsinchu, Taiwan). The sample amount containing 150 µg of proteins was transferred into a 1.5 mL microcentrifuge tube and diluted with water to a final volume of 100 µL. Using the ReadyPrep 2-D Cleanup Kit, the protein pellet was obtained and resuspended by adding 300 µL of rehydration sample buffer (ReadyPrep 2-D Starter Kit Rehydration/Sample Buffer, Bio-Rad, Warsaw, Poland). The supernatants were applied directly to immobilized pH gradient (IPG) stripes (17 cm, pH 3–10, linear pH gradient, Bio-Rad, Warsaw, Poland).

### 4.4. Two-Dimensional Gel Electrophoresis (2DE)

After first-dimensional separation with 12 h of gel rehydration and isoelectric focussing for a total 60 kVh at 20 °C with a current limit of 50 µA per strip (Hoefer IEF100, Hoefer, Inc., Holliston, MA, USA), the second dimension IPG strips were equilibrated according to a procedure described by Klose et al. [12], using an addition of dithiothreitol (2%) and iodoacetamide (2.5%), respectively, for 15 min each. Two-dimensional electrophoretic separation was conducted using 12.5% polyacrylamide gel in BIO-RAD PROTEAN II xi Cell (Bio-Rad, Warsaw, Poland). Vertical separation was performed with 600 V/30 mA/30 W in 0.025 M Tris/glycine pH 8.3 buffer. After electrophoretic separation, proteins were silver-stained, scanned using the Image Scanner III (GE Healthcare, Warsaw, Poland), and processed by Delta2D software (version 4.7, DECODON, Greifswald, Germany) [73]. Statistical analyses were performed to select spots for further proteomic analyses, after manually excluding false-positive and false-negative spots.

### 4.5. Proteomics Analysis

The spots of interest were excised from gel by scalpel, transferred into microtubes, washed with water, and destained. Reduction with dithiothreitol and alkylation with iodoacetamide were conducted. Gel pieces were covered with a trypsin (Trypsin Gold, Promega, Madison, WI, USA) solution containing 50-mM ammonium bicarbonate, and placed in an incubator for overnight digestion at 37 °C. Peptides were then extracted from gels using 50 μL of acetonitrile (ACN) (Merck, Poznań, Poland):water (H_2_O):trifluoroacetic acid (TFA) (Merck, Poznań, Poland) (50:45:5) solution. Extraction was performed using an ultrasonic bath at room temperature, and was repeated three times (each step lasted 15 min). Extracts were collected and concentrated in the CentriVap (Labconco; local seller A.G.A Analitical, Warsaw, Poland). Obtained peptides pellets were dissolved in 10 µL of 0.1% trifluoroacetic acid, and purified with Sample Prep Pipette Tips (ZipTip 0.2 μL C18 Millipore, Merck, Poznań, Poland), according to the standard procedure [74].

### 4.6. MALDI-TOF MS Analysis

MALDI-TOF MS analysis was performed according to a previously proposed procedure [13] with some modifications. Briefly, 1 μL of purified peptide sample was spotted on an AnchorChip MALDI plate. Next 1 μL of α-cyano-4-hydroxycinnamic acid (HCCA) (Bruker, Bremen, Germany) matrix solution was pipetted on the dry peptide sample. Simultaneously, 0.5 µL of a peptide standard was applied to the calibration fields (Peptide Calibration Standard II, Bruker, Bremen, Germany), which was also covered with matrix solution. Mass spectra were recorded in active positive reflector mode within the 700–4000 *m*/*z* range using an Ultraflextreme MALDI TOF/TOF (Bruker, Bremen, Germany) spectrometer and the flexControl 3.3 (Bruker, Bremen, Germany) software. Collected spectra were smoothed using the Savitzky–Golay method, baseline corrected (Top Hat baseline algorithm), and the list of peaks for the signal-to-noise ratio of >3 was generated using the flexAnalysis 3.0 software (Bruker, Bremen, Germany). After the removal of impurities, which originated from trypsin and environmental pollution, the final peak list was transferred to BioTools 3.2 (Bruker, Bremen, Germany), and compared to Mascot 2.2 software (Matrix Science, Boston, MA, USA) using the Swiss-Prot database (www.uniprot.org) restricted to “bony vertebrates” taxonomy. Other parameters were set as follows: maximum error in MS was 0.3 Da, obligatory modification–carbamidomethylation of cysteine, possible modifications included the oxidation of methionine, phosphorylation of serine and threonine, dioxidation of methionine, and protein N-terminal acetylation. Results with a Mascot score above 62 were considered statistically significant (*p* ≤ 0.05); otherwise, the fragment ion spectra of chosen peptides were obtained using the LIFT mode.

### 4.7. Visual and Statistical Analyses

Stained gels were scanned using the Image Scanner III (GE Healthcare, Warsaw, Poland), and processed by Delta2D software (version 4.7; DECODON; Greifswald, Germany). Images were warped—spots of the same protein had the same position across all of the gels in the project—and fused. A fused image responds to the proteome map containing every protein spot obtained in the whole experiment. False positive and false negative spots were manually excluded. To find the expression ratios (quotient of the group means of relative spot volumes), a quantitation table was generated, and statistics were made over normalised volumes. To find the expression ratios (quotient of the group means of relative spot volumes), a quantitation table was generated, and the statistic over normalised volume was made. In this experiment, the mean volume of a given spot in group V was the denominator of the ratio parameter.

Differences in protein expression between the test groups were analysed by one-way ANOVA and a post hoc Tukey comparison test (Graph Pad Prism 5.0, La Jolla, CA, USA) with a *p* value ≤ 0.05 considered significant. Only spots with a significant differential expression between the tumours from the resistant cell lines (FFS1 and FFS3) and the sensitive cell line (FFS5) with a spot intensity ratio higher than 1.5 (up-regulated) or lower than 0.67 (down-regulated) were selected for protein identification.

## 5. Conclusions

Using the 3D in ovo preclinical model of FISS and 2DE followed by MALDI-TOF MS analysis, we identified proteins that may be involved in the chemotherapy resistance of FISS. Three proteins: ANXA5, ANXA3, and MNS1 were identified to be significantly (*p* ≤ 0.05) differentially expressed in doxorubicin-resistant fibrosarcomas in comparison to doxorubicin-sensitive ones. ANXA5 and ANXA3 were up-regulated, and may be correlated with doxorubicin-resistance, and down-regulated MNS1 is a possible indicator of doxorubicin-sensitivity. Moreover, five other proteins were significantly up-regulated, and four were down-regulated in FISS growth from the high doxorubicin-resistant cell line (FFS1) in comparison to the doxorubicin-sensitive cell line (FFS5).

## Figures and Tables

**Table 1:** Significantly (*p* ≤ 0.05) differentially expressed proteins in feline fibrosarcomas from the FFS1 and FFS3 cell lines versus the FFS5 cell line identified by MALDI-TOF MS.

ID	Protein ^1^	*p* Value	Accession Number (UniProtKB)	Species	Score	Match	MW (kDa)^2^	pI ^2^	Modif. ^3^	Seq. Cov. (%)	Ratio I/V^4^	RatioIII/V ^4^
U1	ANXA5	0.0145	P48036	*M. musculus*	194	27	35.79	4.83	C (C); Ox (M)	65	1.51	1.503
U2	ANXA3	0.0119	O35639	*M. musculus*	78	16	36.53	5.50	C (C); Ox (M)	52	1.66	1.74
D1	MNS1	0.0167	Q61884	*M. musculus*	105	14	60.26	6.32	Ac (N-term); C (C) Ox (M); Ac (P N-term)	20	0.29	0.36

^1^ Abbreviations: ANXA5—Annexin A5; ANXA3—Annexin A3; MNS1—meiosis-specific nuclear structural protein 1. ^2^ Listed molecular weights (MW) and pI values correspond to the MASCOT Search Result report correlated with SwissProt database (www.uniprot.org). ^3^ C (C)—carbamidomethylation of cysteine; Ox (M)—oxidation of methionine; Ac (P N-term)—acetylation of protein N-term; Diox (M)—dioxidation of methionine; Ph (ST)—phosphorylation of serine and threonine. ^4^ Ratio—quotient of the group means of relative spot volumes; volume of a given spot in group V is the denominator of the ratio parameter.

**Table 2:** Significantly (*p* ≤ 0.05) differentially expressed proteins in feline fibrosarcomas from the FFS1 cell line versus the FFS5 cell line identified by MALDI-TOF MS analyses.

ID	Protein ^1^	*p* Value	Accession Number (UniProtKB)	Species	Score	Match	MW (kDa) ^1^	pI ^2^	Modif. ^3^	Seq. Cov. (%)	Ratio I/V ^4^
U3	MRP5	0.024	Q9R1X5	*M. musculus*	70	16	162.05	8.80	C (C); Ox (M); Ac (P N-term); Diox (M)	8	2.23
U4	SYDC	0.0289	Q922B2	*M. musculus*	62	12	57.57	6.07	C (C); Ox (M); Ac (P N-term)	32	1.88
U5	SAP18	0.0237	O00422	*H. sapiens*	101	12	17.61	9.38	C (C); Ox (M); Ac (P N-term); Ph (ST)	47	2.83
U6	CBR3	0.0495	Q8K354	*M. musculus*	80	12	31.33	6.15	C (C); Ox (M); Diox (M)	44	2.2
U7	1A1L1	0.0125	Q96QU6	*H. sapiens*	86	13	57.86	6.01	C (C); Ox (M); Ac (P N-term)	26	4.2
D2	ACTA1	0.05	P68134	*M. musculus*	84	26	103.63	5.23	C (C); Ox (M)	29	0.09
D3	ACTN4	0.0283	O43707	*H. sapiens*	120	27	105.37	5.25	C (C); Ox (M); Ac (P N-term)	30	0.37
D4	VIME	0.0152	P20152	*M. musculus*	175	29	53.71	5.06	C (C); Ox (M); Ac (P N-term)	52	0.61
D5	TCPB	0.0344	P80314	*M. musculus*	114	12	57.78	5.97	C (C); Ox (M)	29	0.20

^1^ Abbreviations: MRP5—multidrug resistance protein 5; SYDC—Aspartate–tRNA ligase, cytoplasmic; SAP18—Histone deacetylase complex subunit SAP18; CBR3—Carbonyl reductase (NADPH) 3; 1A1L1—1-aminocyclopropane-1-carboxylate. Synthase-like protein 1; ACTA1—α actin 1; ACTN4—α actinin 4; VIME—vimentin; TCPB—T-complex protein 1 subunit β. ^2^ Listed molecular weights and pI values correspond to the MASCOT Search Result report correlated with SwissProt database (www.uniprot.org). ^3^ C (C)—carbamidomethylation of cysteine; Ox (M)—oxidation of methionine; Ac (P N-term)—acetylation of protein N-term; Diox (M)—dioxidation of methionine; Ph (ST)—phosphorylation of serine and threonine. U—up-regulated; D—down-regulated. ^4^ Ratio—quotient of the group means of relative spot volumes; volume of a given spot in group V is the denominator of the ratio parameter.

## Abbreviations

2DE	Two-dimensional gel electrophoresis
5-FU	5-fluorouracil
ABC	Adenosine triphosphate-binding cassette
Ac (P N-term)	Acetylation of protein N-term
ACN	Acetonitryle
ACTA1	Actin 1
ACTN4	Actinin 4
ANXA3	Annexin A3
ANXA5	Annexin A5
ATP	Adenosine triphosphate
C (C)	Carboamidomethylation of cysteine
CAM	Chorioallantoic membrane
Diox (M)	Dioxidation of methionine
DMEM	Dulbecco’s Modified Eagle’s Medium
FISH	Fluorescence in situ hybridization
FISS	Feline injection-site sarcoma
IC50	The half maximal inhibitory concentration
IPG	Immobilized pH gradient
MALDI-TOF MS	Matrix-assisted laser desorption ionization-time-of-flight-mass-spectrometry
MDR	Multidrug resistance
MNS1	Meiosis-specific nuclear structural protein 1
MS	Mass spectrometry
Ox (M)	Oxidation of methionine
MW	Molecular weight
Ph (ST)	Phosphorylation of serine and threonine
pI	Isoelectic point
RT-PCR	Reverse transcription-polymerase chain reaction
SCP1	Synaptonemal complex protein 1
Sp17	Sperm protein 17
TFA	Trifluoroacetic acid
TRIS	Tris(hydroxymethyl)aminomethane
